# Study of Agave Fiber-Reinforced Biocomposite Films

**DOI:** 10.3390/ma12010099

**Published:** 2018-12-29

**Authors:** Cindu Annandarajah, Peng Li, Mitchel Michel, Yuanfen Chen, Reihaneh Jamshidi, Alper Kiziltas, Richard Hoch, David Grewell, Reza Montazami

**Affiliations:** 1 Agricultural and Biosystems Engineering, Iowa State University, Ames, IA 50011, USA; cindu@iastate.edu (C.A.); mmmichel@iastate.edu (M.M.); 2 Department of Mechanical Engineering, Iowa State University, Ames, IA 50011, USA; pengnova@iastate.edu; 3 College of Mechanical Engineering, Guangxi University, Nanning 530004, China; yuanfenchen@gxu.edu.cn; 4 Department of Mechanical Engineering, University of Hartford, West Hartford, CT 06117, USA; jamshidi@hartford.edu; 5 Ford Motor Company, Dearborn, MI 48120, USA; akizilt1@ford.com; 6 Diageo, London NW10 7HQ, UK; Richard.Hoch@diageo.com; 7 Industrial and Manufacturing Engineering, North Dakota State University, Fargo, ND 58102, USA; david.grewell@ndsu.edu

**Keywords:** agave fiber, polyethylene, polypropylene, thermoplastic, biocomposites, mechanical properties

## Abstract

Thermoplastic resins (linear low-density polyethylene (LLDPE), high-density polyethylene (HDPE), and polypropylene (PP)) reinforced by different content ratios of raw agave fibers were prepared and characterized in terms of their mechanical, thermal, and chemical properties as well as their morphology. The morphological properties of agave fibers and films were characterized by scanning electron microscopy and the variations in chemical interactions between the filler and matrix materials were studied using Fourier-transform infrared spectroscopy. No significant chemical interaction between the filler and matrix was observed. Melting point and crystallinity of the composites were evaluated for the effect of agave fiber on thermal properties of the composites, and modulus and yield strength parameters were inspected for mechanical analysis. While addition of natural fillers did not affect the overall thermal properties of the composite materials, elastic modulus and yielding stress exhibited direct correlation to the filler content and increased as the fiber content was increased. The highest elastic moduli were achieved with 20 wt % agave fiber for all the three composites. The values were increased by 319.3%, 69.2%, and 57.2%, for LLDPE, HDPE, and PP, respectively. The optimum yield stresses were achieved with 20 wt % fiber for LLDPE increasing by 84.2% and with 30 wt % for both HDPE and PP, increasing by 52% and 12.3% respectively.

## 1. Introduction

Over the past decades, conventional petroleum-based plastics have led to environmental issues and sustainability concerns [1,2]. The abundance and sustainability of renewable fillers and reinforcements make them attractive to the polymer industries, especially for automotive, packaging, and construction applications [1,3,4,5]. Also, from the thermomechanical standpoint, bio-based composites were either similar or superior to their petroleum-based equivalents. 

Natural plant fibers have experienced rapid growth in the bio-renewable marketplace in recent years. They have gained various advantages compared to traditional fibers, including lower density, lower cost, prevalence, ease of processing, and low environmental impact [6,7]. Natural fiber reinforcements, such as bast (jute, flax, and hemp); leaf (abaca, sisal); seed (coir); core (kenaf); grass; reed; and wood (lignin and sludge), have been widely reported in the past decades [1,8,9,10]

Recently, plant-based byproducts, such as agave fibers, have received considerable attention from industry. As a leading automobile manufacturing enterprise, Ford has researched the use of sustainable materials for their vehicles since 2000. Currently, Ford uses eight bio-based materials in their vehicles, including soy foam, kenaf fiber, cellulose, wood, coconut fiber, rice husk, castor oil, and wheat straw. Today, Ford is exploring agave-based parts for every single vehicle in their fleet. Agave fiber comes from *Agave Americana*, belonging to the *Agavaceae* family, and the fibers used are a co-product of agave tequila production [11]. Similar to other natural fibers, agave fibers are light, low cost, and reproducible [12]. Despite many desirable properties of agave fiber-based composites, there is only limited published information on agave fiber-based polymer composites. Singha and Rana studied the effects of different fiber concentrations on mechanical properties of polystyrene (PS)/agave-fiber composites and revealed that PS composites reinforced with 20% fiber loading by weight exhibited the best mechanical properties [13]. In another study, Pérez-Fonseca investigated the effect of hybridization of two natural fibers (pine/agave) on the mechanical properties of high-density polyethylene-(pine/agave) based composites and observed that the addition of agave fibers improved the mechanical properties of the composites [14]. Mendizábal’s group studied the effect of agave fiber content in the thermal and mechanical properties of biocomposite [15]. 

Several fiber pretreatment methods, including chemical treatment (alkali, maleated polyethylene, acid, methyl methacrylate), and plasma treatment, have been reported to improve the agave fiber reinforcement effect [6,12,16,17,18]. Alternatively, whether the raw fiber could enhance the mechanical properties of polymers as fillers effectively is also of interest, especially in the automobile manufacturing enterprise. Applications of plant-based byproducts effectively without further chemical treatment have extensive environmental and economic impacts. Moscoso and his colleagues reported that the specific stiffness and strength of PS/agave-fiber composites and foams were enhanced with increasing fiber content [19]. Zuccarello systematically studied the influence of the variety, age, position, and length of the agave fiber, as well as the fiber extraction method on the reinforcement effect of the agave fiber, aiming to develop high performance agave reinforced biocomposite [20,21].

This study evaluated the application of agave fiber without any surface treatment in order to determine whether raw fiber could effectively enhance the mechanical properties of polymer composites applied in the automotive industry. Linear low-density polyethylene (LLDPE), high-density polyethylene (HDPE), and polypropylene (PP) are chosen as the polymer matrices because they are currently widely applied in automotive vehicles. The properties of biocomposite films containing different fiber loading ratios in LLDPE, HDPE, and PP matrices were investigated in this study. Composite films with various agave fiber contents (up to 30 wt %) were prepared by injection and extrusion molding, and their mechanical and thermal properties were studied. Agave fibers were washed to remove residual sugars prior to testing. Preliminary tests from the washing showed that about 0.8 (g/g) amount of residual sugar per unit of fiber can be removed from the washing step. It is envisioned that these sugars could be used to produce additional co-products through fermentation.

## 2. Materials and Methods

### 2.1. Materials

All matrix materials were used in pellet form. The LLDPE had a melt flow index of 20 g/10 min at 190 °C and a density of 0.925 g/cm^3^ (ExxonMobil Chemical Corporation, Houston, TX, USA). The HDPE had a melt flow index of 20 g/10 min at 190 °C and a density of 0.952 g/cm^3^ (Chevron Phillips Chemical Company LP, TX, USA). The PP had a melt flow index of 20 g/10 min at 230 °C and a density of 0.868 g/cm^3^ (Formosa Plastics Corporation, TX, USA). Agave fibers were obtained from Byogy Renewables, Inc. The fibers ranged from 3 to 7 mm in length and were used as received.

### 2.2. Preparation of Agave Fibers

Agave fibers and water were placed in a 70-liter tank at a 2:3 (solid: liquid) volume ratio. The solution was heated to 70 °C and stirred for 24 h, after which the fibers were removed with a filter. This wash cycle was repeated 6 times. The agave fibers were then laid onto drying trays and dried at 105 °C for 18–24 h. The thickness of the fiber layer was less than 1.5.

### 2.3. Preparation of Biocomposite Film

**Figure 1:**
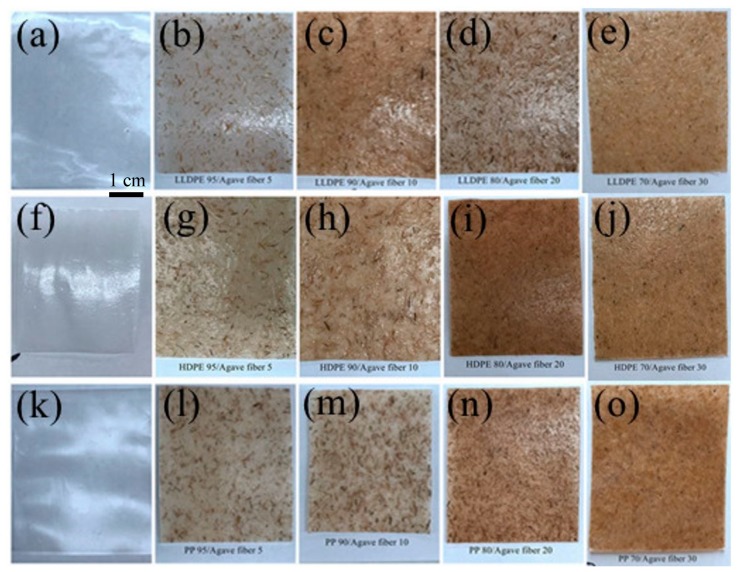
Images of agave fiber reinforced thermoplastic composite films. (**a**–**e**), LLDPE reinforced with agave fiber at 0 wt %, 5 wt %, 10 wt %, 20 wt % and 30 wt %; (**f**–**j**), HDPE reinforced with agave fiber at 0 wt %, 5 wt %, 10 wt %, 20 wt % and 30 wt %; (**k**–**o**), PP reinforced with agave fiber at 0 wt %, 5 wt %, 10 wt %, 20 wt % and 30 wt %.

The various polymers and agave fibers were blended in a Leistritz 27 mm co-rotating twin-screw extruder with a barrel – l/d ratio of 25:1. The extrusion temperatures for the compounding process are detailed in Table 1. In general, polymer matrix/agave fiber compositions with 0, 5, 10, 20, and 30 wt % fiber-content were compounded at a screw speed of 225 RPM. After compounding, the extrudes were pelletized. Subsequently, the pellets were placed into a Brabender plasticorder 19 mm single screw extruder with a barrel – l/d ratio of 25:1 to produce films at a screw speed of 75 RPM. Table 1 details the processing temperatures from the feeder to the die for creating films. Figure 1 shows images of film samples with various fiber content.

### 2.4. Characterization

#### 2.4.1. Scanning Electron Microscopy (SEM)

The morphology of the prepared fibers and the cross-sections of the biocomposite films were investigated using SEM (NeoScopo, JCM-6000 Benchtop SEM) (Peabody, MA, USA). Prior to SEM measurements, the samples were frozen with liquid nitrogen, then fractured along the axis perpendicular to the extrusion direction, to prepare cross-sections. 

#### 2.4.2. Mechanical Analysis

Tensile test was conducted with on dynamic mechanical analyzer (DMA) (DMA-1, Mettler Toledo). All measurements were performed at room temperature (25 °C) at a force rate of 0.5 N/min. Fibers were used as received for single fiber tensile tests and dimensions were measured and taken into account for stress and strain calculations. All composites were 10 mm × 2 mm (length × width). The thickness of the film was measured at three positions for each sample to count for potential variations due to the extrusion conditions. The thickness of the biocomposite films with LLDPE, HDPE and PP matrices ranged from 0.41 to 0.63 mm, 0.34 to 0.54 mm, and 0.31 to 0.47 mm, respectively. ASTM D882-18 Standard Test Method for Tensile Properties of Thin Plastic Sheeting and ASTM D3822/D3822M-14 Standard Test Method for Tensile Properties of Single Textile Fibers were followed while performing the tensile testing. 

#### 2.4.3. Differential Scanning Calorimetry (DSC)

DSC was conducted under ambient conditions (Polymer DSC, Mettler Toledo). The composite samples of both LLDPE and HDPE with agave fiber were heated from 25 °C to 150 °C at a rate of 10 °C/min. The composite samples of PP/agave fiber were heated from 25 °C to 200 °C at a rate of 10 °C/min.

#### 2.4.4. Fourier-Transform Infrared (FT-IR) Spectroscopy

FT-IR spectra of bio-filler reinforced thermoplastic films were recorded in the range of 4000–650 cm^−1^ (Frontier Optica FT-IR spectrometer equipped with UATR accessory, Perkin-Elmer) (Waltham, MA, USA).

## 3. Results and Discussion

### 3.1. Morphology

**Figure 2:**
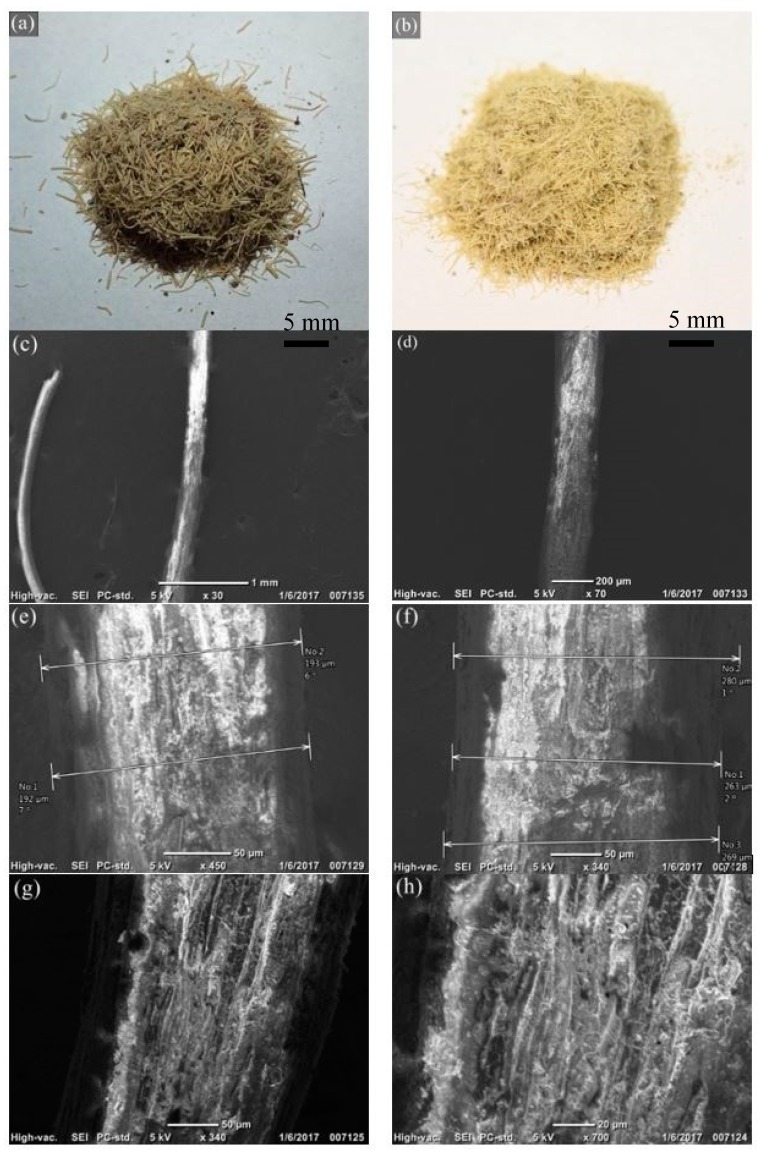
Agave fibers: (**a**) before and (**b**) after washing treatment; SEM images of agave fibers: (**c**) and (**d**): at low magnification showing individual fibers, (**e**) and (**f**) variations in fibers’ diameter, and: (**g**) and (**h**): surface roughness of agave fiber.

Figure 2 shows the images of fiber before and after washing and also the SEM image of a single agave fiber. Figure below shows the raw agave fibers before (a) and after (b) washing. The color of the agave fibers is lighter after washing compared to the unwashed fibers, because the residual sugar in the fiber and the dust on the surface was washed. The washing pretreatment could improve the thermal stability of agave fiber during the processing of composites [2]. Agave fiber has a visibly rough surface and its diameter varies. During the compounding process, the fibers undergo a certain level of thermomechanical damage. In general, the shorter the fiber, the lower the mechanical resistance [22].

**Figure 3:**
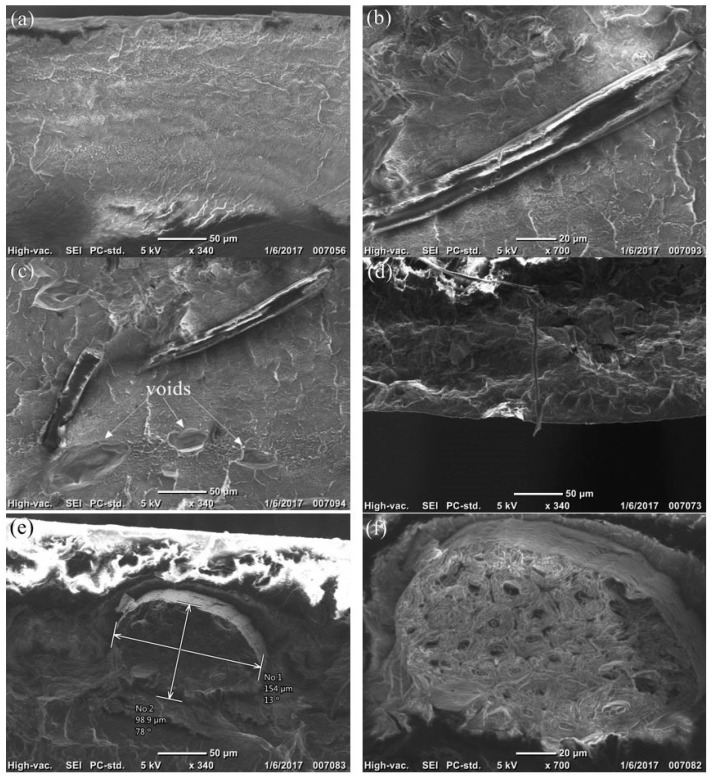
Cross-sectional SEM micrographs of PP/agave fiber films: (**a**) PP control group, (**b–f**) PP: agave fiber 80:20 wt %.

Figure 3 shows SEM cross-sectional images of PP: agave fiber; the morphology of the cross-section of LLDPE and HDPE with agave fiber were similar. Figure 3a shows the control film (PP), while Figure 3b-f show the variations of fibers in the polymer matrix for 80:20 wt % PP-agave composite film. This specific loading level was selected for morphological tests as it has the best mechanical properties overall. In Figure 3c, it can be seen that there are three voids at the bottom part of the image. These voids are likely water vapor bubbles. One explanation for their presence is that although those pellets went through the drying process for more than 24 h, there was still a small amount of moisture in the pellets. The moisture boiled during the extrusion process and caused the formation of voids. Another possibility is that the chemical and polarity differences between polymers and fibers in composites as the polymer is hydrophobic while the fibers are hydrophilic. These differences result in low compatibility between the materials generating voids or gaps at the interface [15,23,24]. Cross-section of an agave fiber at different magnifications are shown in Figure 3e,f. 

### 3.2. FT-IR Analysis of Composite Films

**Figure 4:**
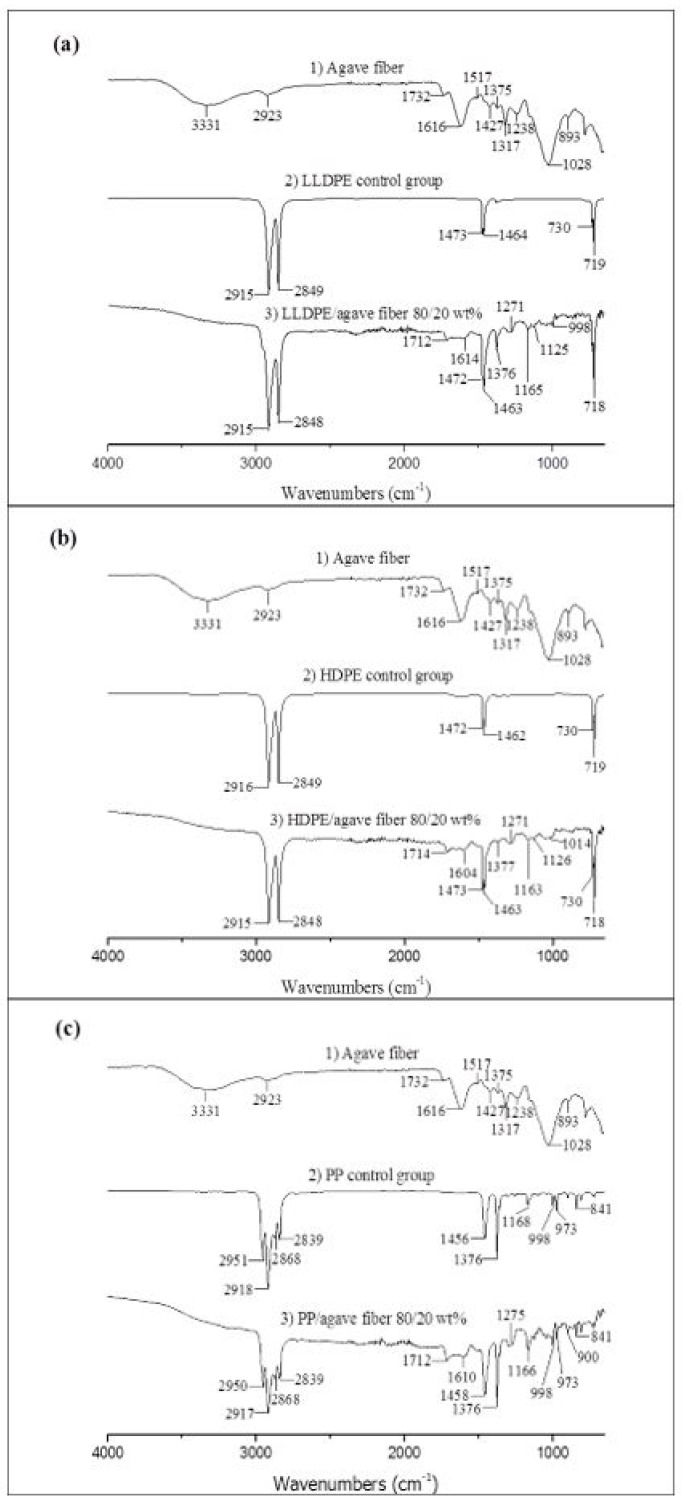
FT-IR spectra of agave fiber reinforced thermoplastic-based composite films: (**a**) LLDPE with agave fiber; (**b**) HDPE with agave fiber; (**c**) PP with agave fiber.

FT-IR spectroscopy was used to examine the interactions between the thermoplastic matrix and the agave fibers. Figure 4a shows the FT-IR spectra of pure agave fibers, LLDPE control group, and 80:20 wt % LLDPE: agave fiber composite. FTIR spectrum of pure agave fiber showed a broad peak at 3331 cm^−1^, which correlates to the stretching vibrations of hydroxyl groups from the cellulose in the agave fiber. The peak at 2923 cm^−1^ was assigned to C–H group. The peak at 1732 cm^−1^ was corresponding to the C=O group of hemicellulose, waxes, pectin, and lignin [25]. The peak at 1616 cm^−1^ was due to H–O–H group stretching of absorbed moisture and for lignin C-H deformation. The peak at 1517 cm^−1^ was due to the lignin aromatic ring vibration and stretching. The milder peaks at 1375 cm^−1^ to 1427 cm^−1^ were attributed to –CH, –CH_2_, or –CH_3_ groups [6]. The peak at 1317 cm^−1^ was due to the –CH group from the cellulose. The peak at 1238 cm^−1^ was assigned to C–O–C and C=O groups of lignin [26]. The peak at 1028 cm^−1^ was attributed to C-O stretching vibrations of the cellulose. The peak at 893 cm^−1^ was due to –β glycosidic linkage of the agave fiber [6].

The LLDPE control group showed peaks at 2915–2849 cm^−1^, 1473–1463 cm^−1^, and 730–719 cm^−1^. These variations where the peaks at 2915–2849 cm^−1^, 1473–1463 cm^−1^, and 730–719 cm^−1^, are assigned to the symmetrical stretching vibration of the C–H bonds. However, the LLDPE composites reinforced with agave fibers exhibited additional peaks: (1) a broad peak between 3600 cm^−1^ and 3200 cm^−1^ was due to the –OH group in the cellulose of agave fiber; (2) the peak at 1712 cm^−1^ was assigned to C=O group of hemicellulose; (3) the peak at 1614 cm^−1^ was due to H–O–H stretching; (4) the peak at 1376 cm^−1^ was attributed to –CH_2_– group; (5) the peak at 1271 cm^−1^ was due to C–O–C stretching; (6) the milder peaks at 1165 cm^−1^ and 1125 cm^−1^ were attributed to O–C–O asymmetric stretching of the cellulose; (7) the peak at 998 cm^−1^ was assigned to C–O stretching vibrations of the cellulose.

Figure 4b shows the spectra of pure agave fiber, an HDPE control group, and an 80:20 wt % HDPE: agave fiber composite. The results for HDPE composites were similar to those for the LLDPE composites. Compared to the neat HDPE, there were several additional peaks exhibited in the composites. The explanations of those additional peaks were the same as the LLDPE composites.

Figure 4c shows the spectra of agave fiber, neat PP, and an 80:20 wt % PP: agave fiber composite. Both neat PP and the PP composites showed four peaks in the wavenumber range between 3000 cm^−1^ and 2800 cm^−1^. The peaks at 2950 cm^−1^ and 2868 cm^−1^ were assigned to –CH_3_ asymmetric and symmetric stretching vibrations, respectively; the peaks at 2918 cm^−1^ and 2839 cm^−1^ were caused by –CH_2_ asymmetric and symmetric stretching vibrations, respectively [27,28,29]. The peak at 1456 was assigned to –CH_2_– or –CH_3_ groups, while the peak at 1376 cm^−1^ was caused by –CH_3_ groups. Various small peaks also appeared in the wavenumber range between 1200 cm^−1^ and 700 cm^−1^: the peak at 1168 cm^−1^ was attributed to C–C, –CH_3_ and C–H groups. The peak at 998 cm^−1^ was assigned to –CH_3_ groups, while the peak at 973 cm^−1^ was attributed to –CH_3_ or C–C groups. The peak at 841 cm^−1^ was assigned to the –CH_2_– group [29]. However, the PP composites reinforced with agave fibers showed additional peaks: (1) a broad peak between 3600 cm^−1^ and 3200 cm^−1^ was due to the –OH group in the cellulose of agave fiber; (2) the peak at 1712 cm^−1^ was assigned to C=O group of hemicellulose; (3) the peak at 1610 cm^−1^ was due to H–O–H stretching; (4) the peak at 1376 cm^−1^ was attributed to –CH2– group; (5) the peak at 1275 cm^−1^ was due to C–O–C stretching; (6) the peak at 1166 cm^−1^ was attributed to O–C–O asymmetric stretching of the cellulose; (7) the peak at 998 cm^−1^ was assigned to C–O stretching vibrations of the cellulose; (8) the peak at 900 cm^−1^ was due to –β glycosidic linkage of the agave fiber.

In general, the FT-IR spectra did not identify the production of chemical bonds at the interface between the polymer matrix and the fibers in either LLDPE, HDPE, or PP composites. 

### 3.3. Mechanical Properties

**Figure 5:**
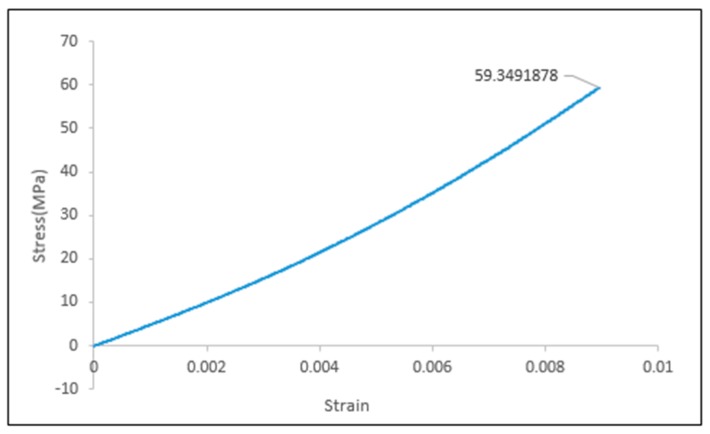
Elastic modulus (E) region of biocomposite films from stress vs. strain graph.

A single fiber tensile test using the ASTM method was performed and the tensile modulus of agave fiber was 6778 ± 823 MPa, which is in agreement with the previously reported value for tensile modulus in literature (15). A typical stress-strain curve for agave fiber is presented in Figure 5. It can be observed that the fiber is still in the linear region at 60 MPa stress, which is far beyond the yield stress of the control films.

**Figure 6:**
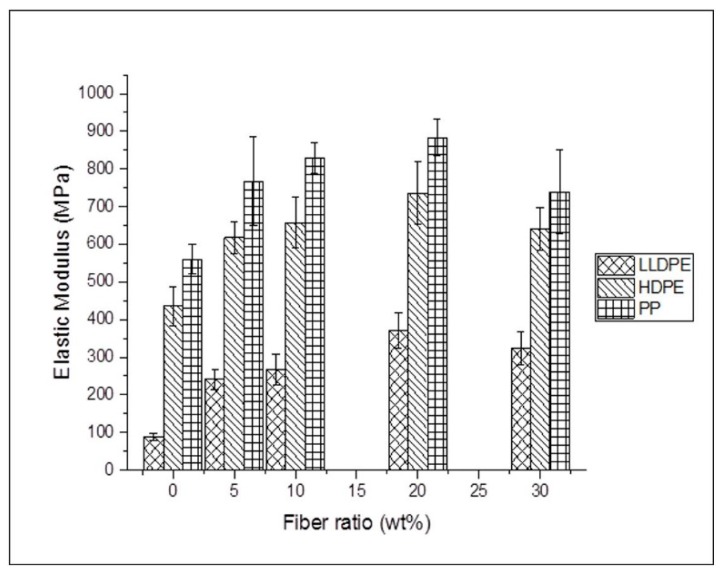
Elastic modulus (*E*) of biocomposites consisting of different fiber content.

**Figure 7:**
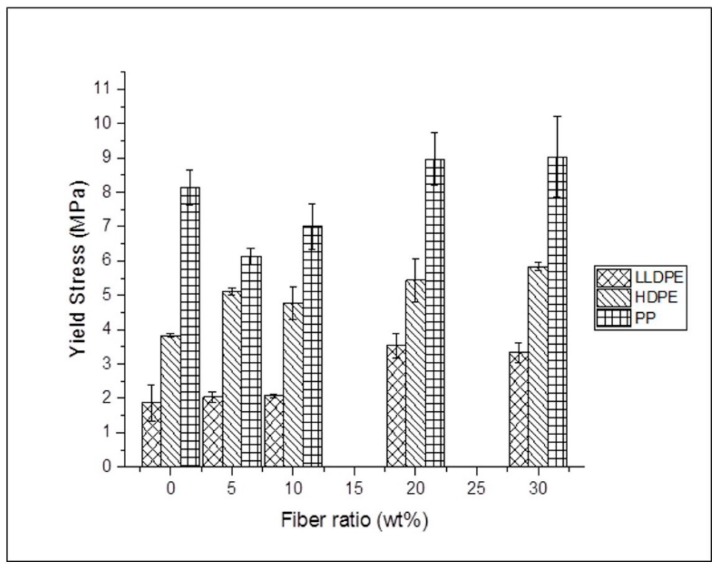
Yield stress (σ) of the composite films and thermoplastic control films.

Figure 6 shows the elastic modulus (*E*) and Figure 7 shows yield stress (σ) of the composite films and thermoplastic control films. The physical and mechanical properties of agave biocomposite films are also summarized in Table 2. The results from Figure 6 and Figure 7 and Table 2 show that the elastic modulus of all the three composites increased as the fiber loading ratio increased and achieved the highest elastic modulus with 20 wt % agave fiber loading. The best achieved elastic modulus was increased by 319.3%, 69.2%, and 57.5%, for LLDPE, HDPE, and PP, respectively. The overall improvement in elastic modulus (E), with increasing agave fiber content can be attributed to the reinforcement effect of agave fiber. A study by Lei et al [30], who investigated recycled high-density polyethylene (rHDPE)/bagasse natural fiber reported similar trend an increase in modulus by about 50 % by adding 30 wt % of bagasses. Hence, incorporation of agave fiber into PP helps in good stress transfer from the matrix to filler and as a result, successfully enhanced the elastic modulus of the composite. The elastic moduli with agave fiber loading at 30 wt % were lower than those at 20 wt %, probably because as fiber content increases, fiber curvature caused by the mixing process decreases the composite property, especially when the fiber length is higher than a critical value [11,31]. 

The results from Figure 7 and Table 2 suggest that the yield strength of agave fiber reinforced LLDPE films increased as fiber loading increased, and the highest yield strength was achieved at 20 wt %, increasing by 84.2%. For the HDPE reinforced with agave fiber, the yield strength was improved compared to that of the neat polymer. The highest yield strength was achieved at 30 wt %, increasing by 52%. For agave fiber reinforced PP composites, the highest yield strength was achieved when the fiber loading was at 30 wt %, increasing by 12.3%. However, the yield strength decreased when the fiber loadings were at 5 wt % and 10 wt %, compared to neat polymer. This might be because when the fiber concentration was lower than a critical value, the amount of fiber is not enough to restrain the matrix, resulting in the development of large, uniformly distributed stresses at low strains [32].

In addition, the specific yield strength was also investigated, the results are shown in Table 2. Compared to neat LLDPE film, the specific yield strength of LLDPE composites were increased as the fiber loading increased. LLDPE composites reinforced with 20 wt % agave fibers loading achieved the highest specific yield strength of 3.55 kN·m/kg, increasing by 94.0%. For HDPE composite, specific yield strengths of all fiber contents were improved compared to the neat polymer. The highest specific yield strength achieved with 30 wt % agave fibers loading, increased by 52.6%. Moreover, compared to the neat PP film, the PP composites reinforced with 20 wt % agave fibers exhibited the highest specific yield strength.

### 3.4. Thermal Analysis

**Figure 8:**
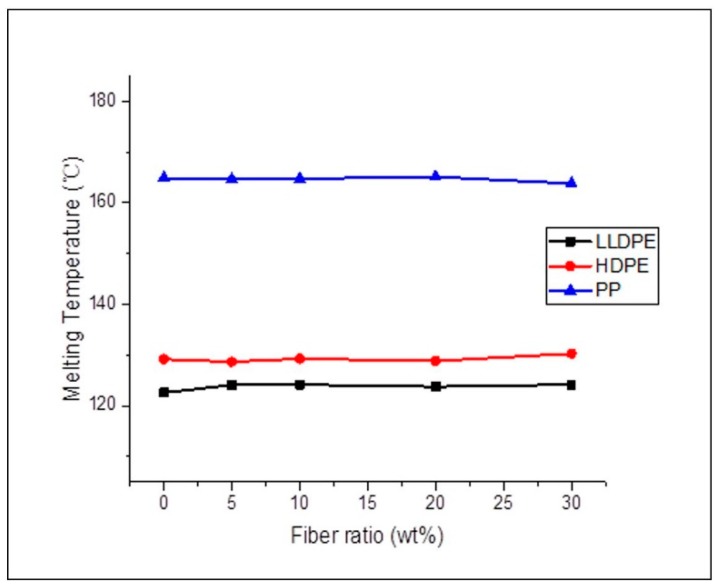
Effect of agave fiber content on melting points of LLDPE, HDPE and PP; no significant variation in T_m_ was observed.

**Figure 9:**
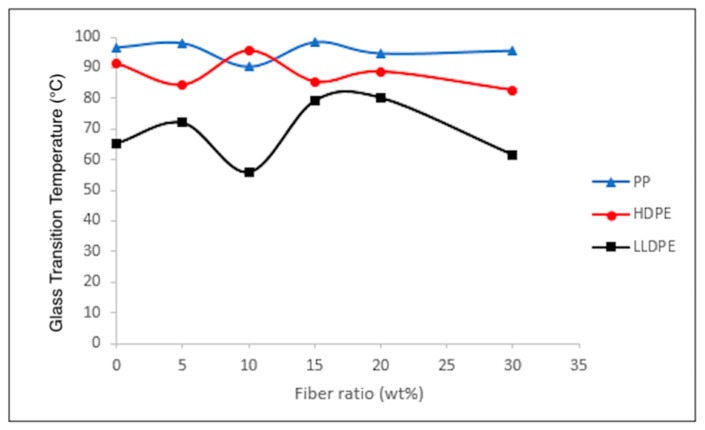
Glass transition temperatures of various composites with different agave fiber contents.

Neat thermoplastics and composites reinforced with agave fibers underwent DSC analysis to determine the changes in the samples’ melting points (*T_m_*). The effect of agave fiber ratio on melting points of LLDPE, HDPE and PP are shown in Figure 8. There were no significant differences in T_m_ between control groups and composites. Crystallinity of the composites were deduced from the DSC glass transition temperature (*T_g_*) curves, Figure 9. The overall crystallinity of the composites is very similar to the neat polymer matrices; however, both increases and decreases in the crystallinity values were observed as the filler ratio increased. This behavior which was observed in all three composites, could result from the fiber acting as either a nucleator or restrictor of the polymer chains, corresponding to an increase or decrease of crystallinity [15,33].

## 4. Conclusions

Biocomposite films made of thermoplastic (LLDPE, HDPE and PP) matrices and agave fiber reinforcement were successfully prepared in order to investigate the effect of raw fiber addition on chemical structure, mechanical and thermal properties. The level of fiber loading was varied between 0 and 30 wt %. Compared to control films, the elastic moduli of composites reinforced with agave fibers exhibited improvement as fiber content increased. The highest elastic modulus was observed when the fiber concentration was at 20 wt %. For LLDPE composites, the highest yield strength was observed when fiber loading was at 20 wt %; for HDPE and PP composites, the highest yield strength was observed when fiber concentration was at 30 wt %. The specific strength of agave fiber reinforced biocomposite film was also investigated and it was observed that the specific yield strength of LLDPE, HDPE, and PP reinforced with agave fiber was improved. The FT-IR spectra suggested that the bonding between fibers and matrices was solely physical and no chemical interaction occurred. Because there were no new chemical bonds created, the thermal properties of the composites were dominated by the matrix resin. The melting temperature of biocomposites did not change significantly by the addition of agave fibers compared to neat thermoplastic films. Although the overall crystallinity of the composites did not change significantly, both an increase and decrease of crystallinity was observed as the fiber ratio was increased. The results from mechanical and thermal characterizations suggested that agave fiber fillers can be used to enhance desired properties in biocomposites and the composites with 20 wt % fiber loading was the optimum formulation.

## Figures and Tables

**Table 1:** The extrusion temperature (°C) parameters of extrusion for compounding and films.

**The extrusion temperature (°C) parameters of extrusion for compounding (twin screw extruder)**												
Heating Zone		1 (feeder)	2	3	4	5	6	7	8	9	10	11(die)
Resin	LLDPE	70	160	165	170	175	180	170	175	170	165	165
	HDPE	70	165	170	175	170	185	185	180	175	170	165
	PP	70	170	195	200	210	215	215	210	205	200	190
**The extrusion temperature (°C) parameters of extrusion for films (single screw extruder)**												
Heating Zone		1 (feeder)	2		3		4		5 (die)			
Resin	LLDPE	90	165		180		175		165			
	HDPE	90	175		185		180		170			
	PP	90	150		190		180		180			

**Table 2:** Mechanical properties of biocomposite films.

	Fiber Ratio (wt %)	Elastic Modulus (MPa)	Standard Deviation	Yield Stress (MPa)	Standard Deviation	Specific Yield Strength (kN·m/kg)
LLDPE: agave fiber	0	88.4	9.29	1.9	0.51	1.83
	5	241.5	27.16	2.0	0.15	2.13
	10	266.7	41.65	2.1	0.06	2.09
	20	370.7	46.55	3.5	0.35	3.55
	30	324.2	43.64	3.3	0.29	3.23
HDPE: agave fiber	0	435.1	50.83	3.8	0.06	3.99
	5	617.6	42.58	5.1	0.1	5.28
	10	657.6	68.23	4.8	0.47	4.89
	20	736.0	82.06	5.4	0.64	5.34
	30	640.3	56.38	5.8	0.12	5.68
PP: agave fiber	0	560.4	40.04	8.1	0.51	9.33
	5	766.7	118.18	6.1	0.23	6.9
	10	829.7	40.73	7.0	0.65	7.78
	20	882.7	48.31	9.0	0.76	9.63
	30	738.8	111.43	9.1	1.17	9.36
